# Deep learning-based automated detection and multiclass classification of soil-transmitted helminths and *Schistosoma mansoni* eggs in fecal smear images

**DOI:** 10.1038/s41598-025-02755-9

**Published:** 2025-07-01

**Authors:** Prosper Oyibo, Brice Meulah, Tope Agbana, Lisette van Lieshout, Wellington Oyibo, Gleb Vdovin, Jan-Carel Diehl

**Affiliations:** 1 Delft Center for Systems and Control, Delft University of Technology, 2628 CN Delft, The Netherlands; 2 School of Engineering, Cardiff University, Cardiff, CF24 3AA UK; 3 Leiden University Center for Infectious Diseases, Leiden University Medical Center, 2333 ZA Leiden, The Netherlands; 4 Centre for Trans-disciplinary Research for Malaria & Neglected Tropical Diseases, College of Medicine, University of Lagos, Lagos, Nigeria; 5 Faculty of Industrial Design Engineering, Delft University of Technology, 2628 CE Delft, The Netherlands

**Keywords:** Digital microscopy, Deep learning, Object detection, *Schistosoma mansoni*, Soil-transmitted helminths, Parasitic infection, Microscopy, Biomedical engineering, Image processing

## Abstract

In this work, we developed an automated system for the detection and classification of soil-transmitted helminths (STH) and *Schistosoma (S.) mansoni* eggs in microscopic images of fecal smears. We assembled an STH and *S. mansoni* dataset comprising over 3,000 field-of-view (FOV) images containing parasite eggs, extracted from more than 300 fecal smear prepared using the Kato-Katz technique. These images were acquired using Schistoscope—a cost-effective automated digital microscope. After annotating the STH and *S. mansoni* eggs, we employed a transfer learning approach to train an EfficientDet deep learning model, using 70% of the dataset for training, 20% for validation, and 10% for testing. The developed model successfully identified STH and *S. mansoni* eggs in the FOV images, achieving weighted average scores of $95.9\%(\pm 1.1\%)$ Precision, $92.1\%(\pm 3.5\%)$ Sensitivity, $98.0\%(\pm 0.76\%)$ Specificity, and $94.0\%(\pm 1.98\%)$ F-Score across four classes of helminths (*A. lumbricoides*, *T. trichiura*, hookworm, and *S. mansoni*). Our system highlights the potential of the Schistoscope, enhanced with artificial intelligence, for detecting STH and *S. mansoni* infections in remote, resource-limited settings and for supporting the monitoring and evaluation of neglected tropical disease (NTD) control programs.

## Introduction

Intestinal helminths are a group of parasitic worms that primarily reside in the intestines of their hosts, including humans. These infections are a significant public health concern, affecting a substantial portion of the global population, particularly in low- and middle-income countries. The most common intestinal helminth infections are caused by soil-transmitted helminths (STH) such as roundworm (*Ascaris lumbricoides*), whipworm (*Trichuris trichiura*), and hookworm (*Necator americanus* and *Ancylostoma duodenale*)^1^. Also, intestinal schistosomiasis caused primarily by *Schistosoma* (*S*.) *mansoni*, *S. japonicum* and *S. intercalatum*, similarly affect the intestines^2^. Over 1.5 billion people, equating to 24% of the global population, are infected with STH infections^3^ while at least 251.4 million people required preventive treatment for schistosomiasis in 2021^4^. Together, STH infections and schistosomiasis account for over 5 million disability-adjusted life years annually^5^. The highest incidences of STH infections and schistosomiasis are reported in Sub-Saharan Africa, the Americas, China, and East Asia^6^.

The WHO has published a roadmap for STH infections and schistosomiasis for this decade (2020-2030), recognizing the importance of diagnostics in stool samples to achieve elimination targets for these diseases^7,8^. Manual screening of a Kato-Katz (KK) thick stool smear by expert microscopists remains the current standard for monitoring the impact of large-scale deworming programs against STH infections and intestinal schistosomiasis^9^. However, this method requires specialized expertise that must be continually developed and maintained, posing an economic challenge, particularly in remote rural communities^10^. There is also a risk of diagnostic errors and visual health complications among microscopists due to excessive workloads resulting from the low ratio of trained microscopists to samples for analysis in endemic regions^11^.

To address these diagnostic challenges, several low-cost automated digital microscopy devices have been developed and validated for the automated detection of STH infections and intestinal schistosomiasis^12,13^. Among these devices is the Schistoscope^14^, developed by our research group, which is capable of automatically focusing and scanning regions of interest on prepared microscopy slides^15^, as well as performing edge artificial intelligence processing^16^. Validation studies have shown it to be a promising and cost-effective tool for the automatic detection of urogenital schistosomiasis in urine samples collected in field settings^17,18^. Preliminary results also indicate the Schistoscope’s potential for analyzing fecal samples, demonstrated by a human reader’s ability to accurately identify *S. mansoni* and hookworm eggs on images of fecal smears captured using the device^14^.

In this study, we aim to develop an artificial intelligence system that can run effectively on the Schistoscope’s edge computing system for the fully automated detection of STH and *S. mansoni* eggs in KK smear in low-resource settings. Specific contributions of this study include:

1. Development of a robust image dataset of KK smears with STH and *S. mansoni* eggs, along with their annotated ground truth.
2. Development of a deep learning based STH and *S. mansoni* egg detection system for low-resource settings.

## Related work

Recent advancements in automating the detection of STH and *S. mansoni* eggs in human fecal smears have leveraged artificial intelligence techniques, with significant progress in accuracy and applicability. These efforts can be broadly categorized into traditional machine learning approaches, deep learning-based detection and segmentation, and dataset-driven challenges, each contributing to the field while facing distinct limitations.

Early work focused on traditional machine learning methods to identify parasite eggs based on handcrafted features. For instance, Alva et al.^19^ employed a logistic regression model using geometric and brightness features but struggled to differentiate parasites with similar morphologies. Similarly, Khairudin et al.^20^ explored k-NN, SVM, and Ensemble classifiers, incorporating feature extraction techniques like Hu’s invariant moments and Gray Level Co-occurrence Matrix (GLCM). Caetano et al.^21^ advanced this direction by optimizing an AdaBoost classifier with swarm intelligence for detecting *S. mansoni* and other helminth eggs, though limited image datasets constrained their accuracy. These studies highlight the potential of traditional methods but underscore their reliance on robust feature engineering and sufficient data, prompting a shift toward deep learning for more generalized solutions.

Deep learning approaches, particularly convolutional neural networks (CNNs) and object detection frameworks, have significantly improved detection performance by learning complex patterns directly from images. Viet et al.^22^ and Olivera et al.^23^ utilized Faster R-CNN to detect parasite eggs, achieving higher accuracy than traditional methods, though small datasets limited generalization. Huo et al.^24^ and Naing et al.^25^ adopted YOLO-based models (YOLOv5 and YOLOv4-Tiny, respectively), demonstrating improved speed and accuracy, particularly when high-magnification images captured distinct features. Rajasekar et al.^26^ further advanced this trend, showing that YOLOv8 with an SGD optimizer outperformed models like Detectron2 and InceptionV3. For real-time applications, Delas Penas et al.^27^ implemented a tiny YOLO framework, which showed promise for rapid processing but lower accuracy for STH eggs compared to *S. mansoni*. Meanwhile, segmentation-focused studies, such as Libouga et al.^28^ with a modified U-Net and Lim et al.^29^ comparing VGG and ResNet to traditional fuzzy c-Mean clustering, demonstrated deep learning’s superiority in delineating parasite eggs from complex backgrounds.

Innovative pipelines combining detection and classification have also emerged. Dacal et al.^30^ proposed an SSD-MobileNet pipeline for remote analysis of *Trichuris trichiura* eggs in KK samples, while Lee et al.^31^ integrated SSD, U-Net, and Faster R-CNN for comprehensive egg identification and quantification. Lundin et al.^32^ employed sequential CNNs (YOLOv2 for detection and ResNet50 for classification) to identify STH eggs by species, though their system overestimated egg counts compared to manual microscopy, highlighting challenges in calibration. Approaches for smartphones and resource-constrained settings have also been explored, with Yang et al.^33^ developing Kankanet, an ANN-based smartphone application, and Lin et al.^34^ applying MobileNetV2 for egg classification, both constrained by low-quality images or small datasets.

Despite these advancements, dataset limitations remain a critical challenge across studies. Roder et al.^35^ achieved promising results with Deep Belief Networks on a small grayscale dataset, but scalability was limited. Ward et al.^36^ created a large dataset of 7780 KK smear images, yet uneven egg distribution (50% belonging to *A. lumbricoides*) and reliance on high-infection-intensity slides risked biasing their model. Acula et al.^37^ and Nakasi et al.^38^ also noted that insufficiently robust datasets hampered CNN performance, even with architectures like ResNet-50, AlexNet, and GoogleNet.

Collectively, these studies illustrate the field’s progress toward accurate and scalable helminth egg detection while highlighting persistent challenges in dataset quality, image resolution, and model generalization. This work builds on these efforts by addressing dataset robustness and enhancing model accuracy, with a focus on practical deployment in low-resource settings where automated diagnostics are most needed.

**Fig. 1:**
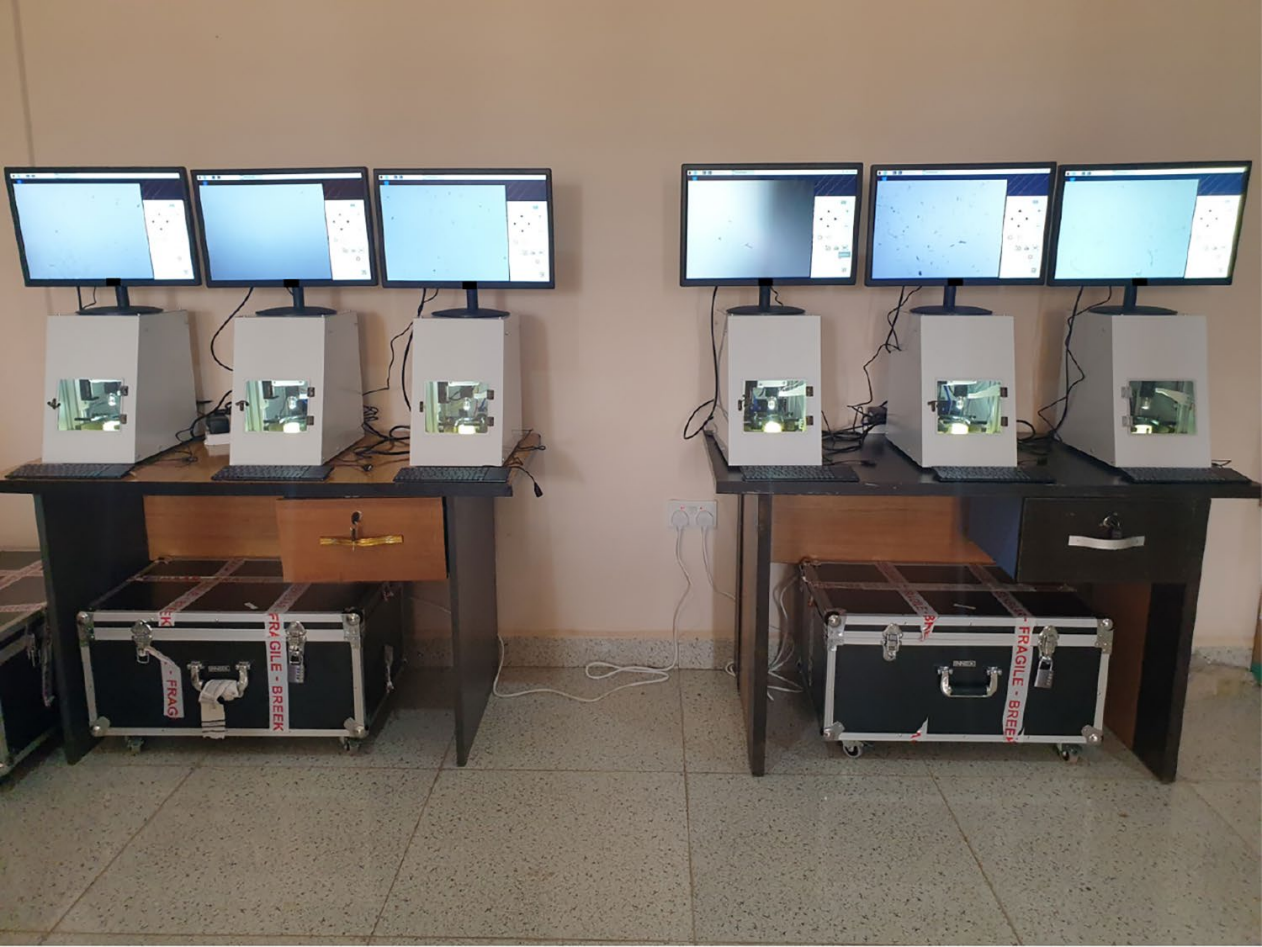
Field laboratory setup, equipped with 6 Schistoscope devices.

## Methodology

### STH and *S. mansoni* dataset

Image acquisition was performed during field studies carried out in the Federal Capital Territory (FCT), Nigeria. Ethical approval for the research was granted by the FCT Health Research Ethics Committee under approval number FHREC/2022/01/102/05-07-22 and the research was performed in accordance with the relevant guidelines and regulations. The project was presented to the NTD Unit of the Public Health Department, FCT Abuja, which then informed the local NTD officer in the selected area councils. Following informed consent, fecal samples were collected from school-age children in sterile 20 mL universal containers. The fecal samples were processed using the standard Kato-Katz technique with a 41.7 mg template^39^. To accelerate data acquisition, we established a field lab equipped with 6 Schistoscope devices (as shown in Fig. 1) to image the processed slides.

The Schistoscope was configured with a 4$\times$ objective lens (0.10 NA). A total of 300 sample slides prepared using the KK stool thick smears technique were registered, resulting in 141,600 FOV images with a resolution of 2028 $\times$ 1520 pixels. The images were screened and manually annotated by expert microscopists, identifying 889 hookworm and 3238 *S. mansoni* eggs present in 3040 FOV images. To obtain a robust dataset for the development of the deep learning model, we combined our registered dataset with the dataset from Ward et al.^40^, which contains FOV images, from over 300 KK freshly prepared stool thick smears, registered with a prototype slide scanner and annotated labels containing 8600 *A. lumbricoides*, 4083 *T. trichiura*, 3623 hookworm, and 682 *S. mansoni*. The combined dataset consists of 10,820 FOV (71.9% adopted from Ward et al.^40^ and 28.1% registered by the Schistoscope ) images with a total of 8600 *A. lumbricoides*, 4082 *T. trichiura*, 4512 hookworm, and 3920 *S. mansoni* eggs as shown in Table 1. FOV images were randomly shuffled and split into three datasets: a training set, a validation set, and a test set. We aimed for a desired split ratio of 70:20:10 for both our created dataset and the Ward et al.^40^ dataset in the combined dataset as shown in Table 2.

**Table 1:** Number of helminth eggs in the datasets.

Dataset	Scanned slides	FOV images		Verified helminth eggs				
		Registered	With eggs	*A. lumbricoides*	*T. trichiura*	hookworm	*S. mansoni*	Total
Ward et al.^40^	272	1,386,186	7780	8600	4083	3623	682	16,990
Present work	300	141,600	3040	0	0	889	3238	4127
Combined	572	1,527,786	10,820	8600	4083	4512	3920	21,117

**Table 2:** Train, validation and test dataset split.

Split set	FOV images	Verified helminth eggs				
		*A. lumbricoides*	*T. trichiura*	Hookworm	*S. mansoni*	Total
Train (70% target)	7953 (69.4%, 30.6%)	6071	2839	3226	3070	15,205
Validation (20% target)	1808 (83.2%, 16.8%)	1646	859	803	466	3774
Test (10% target)	1059 (71.0%, 29.0%)	883	385	483	384	2135
Total	10,820 (71.9%, 28.1%)	8600	4083	4512	3920	21,117

### Deep learning model

EfficientDet^41^ is a state-of-the-art deep learning architecture developed by Google brain team. It is designed to be both fast and accurate across a wide range of computing environments, from mobile devices to servers which makes it suitable for applications such as edge systems with limited computational resource. It builds on EfficientNet, a scalable neural network architecture, by incorporating a novel compound scaling method that simultaneously scales up the resolution, depth, and width of the model, as well as the feature network and the box/class prediction network. Our developed model for the Classification of the STH (i.e., *A. lumbricoides, T. trichiura *and hookworm) and *S. mansoni* eggs, is based on the EfficientDet-D0 architecture, which integrates a Single Shot Detector (SSD) framework with an EfficientNet-B0 backbone. The backbone, EfficientNet-B0, is augmented by a Bi-directional Feature Pyramid Network (BiFPN). The BiFPN is configured to operate across feature levels 3–7 with three iterations and 64 filters, enhancing the model’s ability to fuse features from different resolutions. The model employs a weight-shared convolutional box predictor, which helps in reducing the number of parameters by sharing weights across different layers. This predictor has a depth of 64, utilizes depthwise separable convolutions, and is optimized with SWISH activation and L2 regularization. For classification, the model uses a weighted sigmoid focal loss with parameters $\alpha = 0.25$ and $\gamma = 1.5$, which is particularly effective in dealing with class imbalance by down-weighting the loss assigned to well-classified examples. The localization loss is computed using a weighted smooth L1 loss, balancing the accuracy of bounding box predictions. Both classification and localization losses are normalized by the number of matches and code size to ensure stable training. Multiscale anchors are generated with scales ranging from level 3 to 7, an anchor scale of 4.0, and three aspect ratios (1.0, 2.0, 0.5). This allows the model to detect objects at multiple scales. The model uses an argmax matcher with a threshold of 0.5 for both matched and unmatched cases, ensuring that every ground truth box is assigned to the best-matching anchor. Input images are resized to maintain their aspect ratio within dimensions of 512 $\times$ 512 pixels, with padding added to fit the maximum dimension. The training process includes data augmentation techniques like random horizontal flips and random scaling, cropping, and padding, enhancing the model’s robustness to various image transformations. The model is fine-tuned from a pre-trained EfficientDet-D0 checkpoint trained on the COCO dataset^42^, specifically tailored for detection tasks. A momentum optimizer is used with a cosine decay learning rate schedule, starting at 0.0008 and gradually decreasing over 400,000 steps, with a warmup phase for the first 2500 steps. The model was implemented using the Python TensorFlow library and trained on the Google Colab platform with an A100 GPU, using a batch size of 16.

### Performance measurement

To evaluate the performance of the STH and *S. mansoni* egg classification task, we used precision, sensitivity, specificity, and F1-score. These metrics are mathematically defined as follows:

1 $$\begin{aligned} Precision= & \frac{TP}{TP+FP} \end{aligned}$$

2 $$\begin{aligned} Sensitivity= & \frac{TP}{TP+FN} \end{aligned}$$

3 $$\begin{aligned} Specificity= & \frac{TN}{TN+FP} \end{aligned}$$

4 $$\begin{aligned} F1\text {-}score= & 2 \times \frac{Precision \times Sensitivity}{Precision + Sensitivity}, \end{aligned}$$

where *TP*, *FP*, *TN* and *FN* are True Positive, False Positive, True Negative and False Negative samples respectively.

## Results

**Fig. 2:**
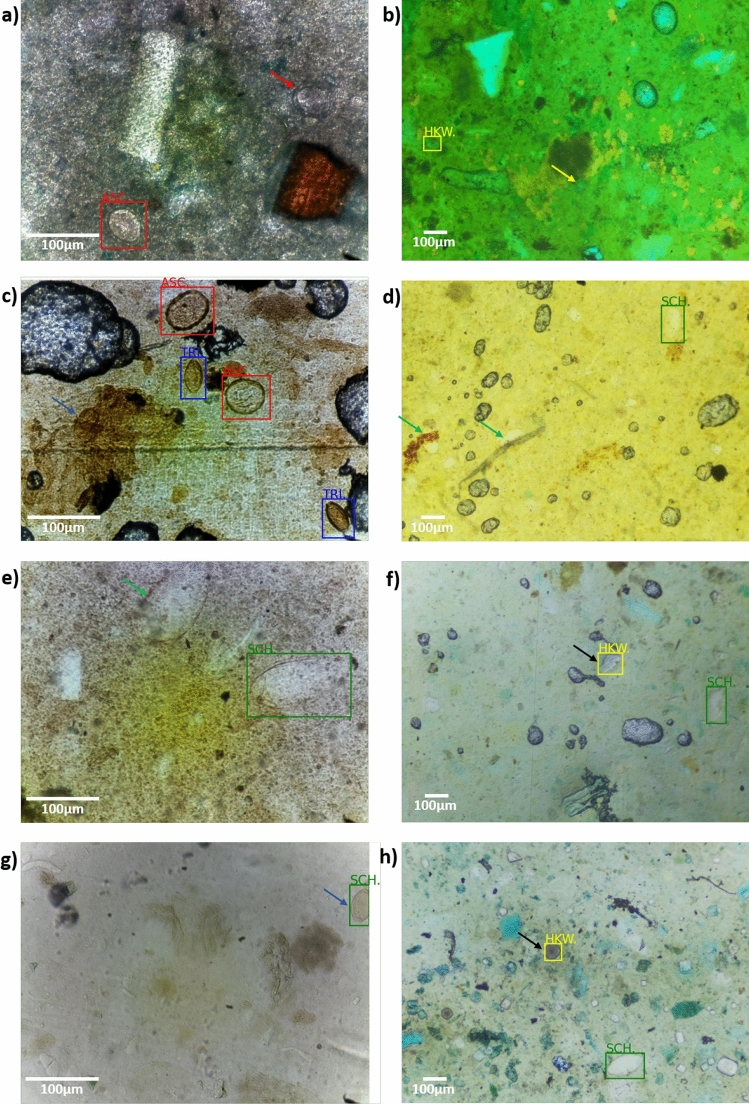
Example images from the combined test dataset. Images (a), (c), (e), and (g) are from Ward et al.^40^, while images (b), (d), (f), and (h) were captured using the Schistoscope. Eggs detected and classified by the deep learning model are highlighted with red, blue, yellow, and green bounding boxes, corresponding to *Ascaris lumbricoides*, *Trichuris trichiura*, hookworm, and *Schistosoma mansoni*, respectively. Arrows indicate missed or misclassified eggs: red, blue, yellow, and green for *A. lumbricoides*, *T. trichiura*, hookworm, and *S. mansoni*, respectively; black arrows mark artifacts incorrectly classified as eggs.

Figure 2 shows the results of images with presence of artifacts in the fecal material which complicates the identification of eggs. Images (a), (c), (e), and (g) originate from Ward et al.^40^, while images (b), (d), (f), and (h) were acquired using the Schistoscope. The eggs detected and classified by the developed deep learning model are enclosed in bounding boxes: red for *A. lumbricoides*, blue for *T. trichiura*, yellow for hookworm, and green for *S. mansoni*. Arrows indicate instances of missed or misclassified eggs using the same color scheme. Black arrows point to artifacts that were incorrectly identified as eggs by the model. The developed model failed to detect *A. lumbricoides*, hookworm, *T. trichiura*, and *S. mansoni* eggs in images (a), (b), (c), and (e), respectively, due to improperly cleared fecal smears. In image (d), two *S. mansoni* eggs were obscured by artifacts and not detected. Artifacts in images (f) and (h) were misidentified as hookworm eggs, and a *T. trichiura* egg in image (g) was incorrectly classified as a *S. mansoni* egg. Differences in egg sizes across the dataset result from varying optical device configurations used for image acquisition (Ward et al.^40^: 10 $\times$ magnification, 0.25 NA; Schistoscope: 4 $\times$ magnification, 0.1 NA). However, these variations in resolution, combined with artifacts and diverse background colors and textures in fecal samples, enhanced the dataset’s robustness and helped mitigate overfitting.

The confusion matrix (shown in Table 3) evaluates the model’s performance in detecting the four classes of helminth eggs. The model exhibited high detection and classification accuracy (shown in Table 4), with precision and sensitivity for *A. lumbricoides* at 0.968 and 0.949, and for *T. trichiura* at 0.943 and 0.951, respectively. hookworm had a precision of 0.949 and sensitivity of 0.878, while *S. mansoni* showed 0.968 precision and 0.878 sensitivity. Our deep learning model, based on EfficientDet-D0, achieves a weighted average precision of 95.9% (± 1.1%), sensitivity of 92.1% (± 3.5%), specificity of 98.0% (± 0.76%), and F1-score of 94.0% (± 1.98%) across the four helminth classes. These metrics confirm the model’s accuracy and reliability in detecting and classifying STH and *S. mansoni* eggs, despite variations in image conditions.

**Table 3:** Confusion matrix.

		AI predictions and performance				
		*A. lumbricoides*	*T. trichiura*	hookworm	*S. mansoni*	False negatives (missed eggs)
Verified ground truth	*A. lumbricoides*	790	1	0	0	42
	*T. trichiura*	1	364	0	0	19
	Hookworm	0	0	424	0	59
	*S. mansoni*	0	0	0	337	47
False positives (background artefacts)		26	22	23	11	–

**Table 4:** Performance metrics.

	*A. lumbricoides*	*T. trichiura*	Hookworm	*S. mansoni*	Weighted average	Standard deviation
Precision	0.968	0.943	0.949	0.968	0.959	0.011
Sensitivity	0.949	0.951	0.878	0.878	0.921	0.035
Specificity	0.972	0.984	0.982	0.992	0.980	0.0076
F-Score	0.959	0.947	0.912	0.921	0.940	0.0198

## Discussion

The World Health Organization (WHO) has outlined Target Product Profiles (TPPs) for diagnostic tools to control STH infections and schistosomiasis, emphasizing affordability, accessibility, and effectiveness in resource-limited settings^43,44^. This study advances these goals through the Schistoscope, a cost-effective automated microscope enhanced with an artificial intelligence (AI) system for detecting and classifying STH and *S. mansoni* eggs. Unlike many prior efforts, our work uniquely integrates edge-computing capabilities, a robust and diverse dataset, and a focus on practical deployment, offering distinct advantages over existing approaches.

The Schistoscope’s design prioritizes affordability and usability, leveraging off-the-shelf components for easy maintenance and scalability in low-resource settings. Its AI-driven system enables automatic focusing, scanning, and egg detection, reducing reliance on skilled microscopists-a critical bottleneck noted in manual KK diagnostics^10^. Compared to earlier automated microscopy systems, such as those by Holmström et al.^12^, which required external computational resources, the Schistoscope’s edge-computing capability allows real-time processing in remote areas without internet connectivity. This contrasts with studies like Dacal et al.^30^, which relied on telemedicine pipelines, limiting their applicability in disconnected settings. Our prior work validated the Schistoscope’s efficacy for *S. haematobium* egg detection^16,18^, and this study extends its utility to STH and *S. mansoni* eggs detection, demonstrating versatility across parasitic diseases.

A key contribution of this work is the development of a comprehensive STH and *S. mansoni* eggs Dataset, comprising 141,600 FOV images from 300 KK slides captured using the Schistoscope’s 4$\times$ objective lens (0.10 NA), with 3040 FOVs containing 889 hookworm and 3238 *S. mansoni* eggs. By augmenting this with the Ward et al.^40^ dataset, we created a combined dataset of 10,820 FOVs with 21,117 eggs across four species (*A. lumbricoides*, *T. trichiura*, hookworm and *S. mansoni*). Unlike Ward et al.’s dataset, which suffered from class imbalance (50% *A. lumbricoides* eggs) and high-infection-intensity bias, our dataset improves balance for *S. mansoni* and incorporates diverse stool samples from 300 individuals. This addresses limitations in prior datasets, such as those used by Roder et al.^35^ and Nakasi et al.^38^, which were constrained by small or grayscale images, enhancing model generalizability.

Our developed model’s performance compares favorably to prior studies. For instance, Viet et al.^22^ and Olivera et al.^23^ used Faster R-CNN but faced generalization issues due to small datasets, while Huo et al.^24^ and Rajasekar et al.^26^ achieved high accuracy with YOLO models but required high-magnification images impractical for low-cost devices. Our model’s performance on lower-magnification (4$\times$) images, combined with edge-computing efficiency, makes it more suitable for field deployment than resource-intensive models like ResNet-50 used by Lundin et al.^32^, which overestimated egg counts. Additionally, unlike smartphone-based solutions like Yang et al.’s Kankanet^33^, which were limited by image quality, the Schistoscope ensures consistent imaging, improving reliability.

Despite these strengths, a limitation of our dataset is the absence of *A. lumbricoides* and *T. trichiura* eggs captured with the Schistoscope, due to their non-prevalence at our study sites. This may bias the model toward hookworm and *S. mansoni* detection in Schistoscope images, a challenge also noted in studies with uneven class distributions^40^. Sensitivity for hookworm (0.878) and *S. mansoni* (0.878) is slightly lower than for other classes, likely due to variability in image sources, but precision (0.949-0.968) and specificity (0.921–0.992) remain high, with low standard deviations indicating robustness. Future work will expand the dataset to include more *A. lumbricoides* and *T. trichiura* eggs and refine annotations to boost sensitivity, explore other deep learning architectures to improve quantification building on insights from medical imaging studies.

This study’s uniqueness lies in its end-to-end solution: a low-cost, AI-enhanced device with a robust dataset and high performance tailored for low-resource settings. While prior works advanced classification, they lacked scalable hardware integration. Our system aligns with WHO TPPs, offering a practical tool for monitoring deworming programs. Evaluating performance at the slide/patient level, as opposed to only image-level metrics, will further ensure clinical reliability.

## Conclusion

In conclusion, the Schistoscope, combined with an AI-based detection system, demonstrates strong potential for accurately detecting STH and *S. mansoni* eggs, aligning with WHO’s vision for affordable and accessible diagnostics in low-resource settings. Our model exhibited high precision, sensitivity, and specificity across all classes, with room for improvement in the detection of hookworm and *S. mansoni* eggs. Expanding the dataset and optimizing model parameters will further enhance performance and generalizability. Overall, the system holds promise for supporting large-scale monitoring and deworming efforts in endemic regions. In future we would like to evaluate the diagnostic performance of the system in a resource limited settings.

## Data Availability

The data used in this study is publicly available for research and development from the following sources: AI4NTD KK2.0 P1.5 STH & SCHm Dataset: https://www.kaggle.com/datasets/peterkward/ai4ntd-p1-5, Hookworm and Schistosoma mansoni Eggs Image Dataset: https://doi.org/10.5281/zenodo.13843815.
